# P2Y6 and P2X7 Receptor Antagonism Exerts Neuroprotective/ Neuroregenerative Effects in an Animal Model of Parkinson’s Disease

**DOI:** 10.3389/fncel.2019.00476

**Published:** 2019-11-08

**Authors:** Ágatha Oliveira-Giacomelli, Carolina M. Albino, Hellio Danny Nóbrega de Souza, Juliana Corrêa-Velloso, Ana Paula de Jesus Santos, Juliana Baranova, Henning Ulrich

**Affiliations:** Neuroscience Laboratory, Department of Biochemistry, Institute of Chemistry, University of São Paulo, São Paulo, Brazil

**Keywords:** purinergic receptors, Brilliant Blue G, P2X7 receptor, MRS2578, P2Y6 receptor, Parkinson’s disease, microglia

## Abstract

Parkinson’s disease (PD) is a neurodegenerative disorder characterized by decreased dopamine bioavailability in the *substantia nigra* and the *striatum*. Taking into account that adenosine-5’-triphosphate (ATP) and its metabolites are intensely released in the 6-hydroxydopamine (6-OHDA) animal model of PD, screening of purinergic receptor gene expression was performed. Effects of pharmacological P2Y6 or P2X7 receptor antagonism were studied in preventing or reversing hemiparkinsonian behavior and dopaminergic deficits in this animal model. P2X7 receptor antagonism with Brilliant Blue G (BBG) at a dose of 75 mg/kg re-established the dopaminergic nigrostriatal pathway in rats injured with 6-OHDA. Selective P2Y6 receptor antagonism by MRS2578 prevented dopaminergic neuron death in SH-SY5Y cells *in vitro* and *in vivo* in the *substantia nigra* of rats injured with 6-OHDA. Moreover, *in vivo* analysis showed that both treatments were accompanied by a reduction of microglial activation in the *substantia nigra*. Altogether, these data provide evidence that antagonism of P2X7 or P2Y6 receptors results in neuroregenerative or neuroprotective effects, respectively, possibly through modulation of neuroinflammatory responses.

## Introduction

Parkinson’s disease (PD) is highly incapacitating and affects nearly 1% of individuals between 65 and 69 years, reaching almost 3% of individuals after 80 years of age (Nussbaum and Ellis, 2003). The pathophysiology of this disease is based on dopaminergic neuron loss in the nigrostriatal pathway and presence of protein aggregates positive for α-synuclein, known as Lewis bodies (Hornykiewicz, 1966). It is known that neurodegeneration of *substantia nigra* results in *striatum* enervation atrophy accompanied by diminished dopamine bioavailability (Hughes et al., 1992). Since occurrence of neurogenesis and presence of multipotent stem cell in ventricular and subgranular zone of hippocampus of the adult brain had been confirmed, several studies arose regarding the possibility of replenishing dopaminergic neuron loss. Therefore, elucidation of mechanisms of induction, integration and survival of newborn neurons has become important.

In neurodegenerative diseases, cell death tends to expand as a consequence of neuroinflammatory processes triggered by the release of biomolecules—such as adenosine-5’-triphosphate (ATP)—called Danger Associated Molecular Patterns (DAMPs), which act as danger signals and recruit microglial cells as representatives of the immune system in the brain. In response to DAMP-induced stimulation, resting microglial cells that broadly populate the central nervous system (CNS) rapidly become reactive, acquiring ameboid morphologies, and migrate to the location of neurodegeneration, as observed in postmortem brains of patients with PD (Pasqualetti et al., 2015). During immune response, DAMPs activate pattern recognition receptors, widely expressed in the CNS by microglial cells, neurons and astrocytes (Kigerl et al., 2014). DAMPs modulate pro-apoptotic and proinflammatory intracellular signaling cascades that propagate the inflammatory response and exacerbate neuronal death in humans and animal models of PD (Wilms et al., 2003; Davalos et al., 2005). This activation exacerbates the release of ATP, which binds to P2X7 receptors and consequently activates NACHT, LRR and PYD domains-containing protein 3 (NALP3) inflammasomes and proinflammatory interleukin secretion (Di Virgilio, 2007). As recently reviewed by Calovi et al. (2019), different purinergic receptors are involved in microglial activation under pathological scenarios, and their activity modulation could interfere with neuroinflammation processes.

Large amounts of ATP released by dying cells into the extracellular space activate P2X7 and possibly other purinergic receptor subtypes, which may exert important roles in PD-related neurodegeneration. P2X7 receptor signaling induced by high extracellular ATP concentration in pathological scenarios may trigger apoptosis through membrane pore formation (Burnstock, 2004). P2X7 receptor antagonists, including A-438079 and Brilliant Blue G (BBG), promoted preventive or restorative effects on dopaminergic neuron deficits in animal models of PD (Marcellino et al., 2010; Carmo et al., 2014; Ferrazoli et al., 2017). Choi et al. (2009) used single-cell real time polymerase chain reaction (RT-PCR) for demonstrating that dopaminergic neurons do not express the P2X7 receptor, which raises the hypothesis of glial modulation of the protective effect exerted by BBG. Corroborating this hypothesis, co-localization of glial cells with the P2X7 receptor was observed in immunohistochemical analysis of rat *substantia nigra* (Marcellino et al., 2010).

Besides P2X7 receptors, metabotropic purinergic receptors, including the P2Y6 subtype have been suggested to function in neuroprotection and neuroregeneration (Calovi et al., 2019). A recent study showed that *P2Y6* receptor gene expression was increased in SH-SY5Y human neuroblastoma cells, an *in vitro* model of dopaminergic neurons, when subjected to an insult with the neurotoxin 1-methyl-4-phenylpyridinium (MPP^+^; Qian et al., 2018). In addition, pharmacological antagonism or si-RNA mediated knock down of this receptor counteracted MPP^+^-induced cell death by reducing production of reactive oxygen species (Qian et al., 2018). In cell death processes *in vivo*, release of uridine-5’-diphosphate (UDP) by apoptotic cells and consequent activation of P2Y6 receptor induced the production of cytokines by microglial cells and their phagocytic activation, indicating that this receptor subtype may be involved in the inflammatory response of neurodegenerative diseases (Kim et al., 2011). Peripheral blood mononuclear cells from patients with PD below the age of 80 years expressed high levels of P2Y6 receptors, and the use of an *in vitro* microglial response model corroborated the hypothesis of microglia involvement in the neuroinflammatory effect (Yang et al., 2017).

In the present study, we analyzed *P2X7* and *P2Y6* receptor gene expression in 6-hydroxydopamine (6-OHDA)-induced lesion in rats. Based on previous reports on the involvement of these receptors in anti-inflammatory processes, the P2X7 receptor antagonist BBG and the selective P2Y6 receptor antagonist MRS2578 were used separately *in vivo*.

## Materials and Methods

### Animals

Male Sprague–Dawley rats, 60 days of age at the beginning of treatment, were housed by the animal facility of the Institute of Chemistry-University of Sao Paulo, with unlimited access to food and water and light/dark cycle of 12:12 h. This study was carried out in accordance with the principles of the Basel Declaration and recommendations of Guidelines of the Brazilian College of Animal Experimentation and NIH Guide for Care and Use of Laboratory Animals. The protocol was approved by the local ethics’ committee (certificates 15/2013, 04/2014, 57/2017, 105/2018).

#### Induction of Parkinson’s Disease in Animal Model

Unilateral lesion of the nigrostriatal pathway was performed according to Ferrazoli et al. (2017). Briefly, animals were anesthetized and placed in a stereotaxic frame (KOPF, Los Angeles, CA, USA). A punctual incision was made in the skull using a drill. Two microliters of a freshly prepared 6-OHDA (Sigma-Aldrich, St. Louis, MO, USA) at the concentration of 7 μg/μl dissolved in 0.9% saline solution containing 0.02% ascorbic acid (Sigma-Aldrich) was injected into the medial forebrain bundle of the right brain hemisphere (Annett et al., 1992) using a 30G needle coupled to a Hamilton syringe, following stereotactic coordinates from the Bregma: AP −4.4; ML −1.2; DV −8.2 (Paxinos and Watson, 2013).

#### Behavioral Test: Apomorphine-Induced Rotational Behavior

In order to confirm 6-OHDA model induction, animals were submitted to rotational tests after 1 week of the nigrostriatal pathway injury, and animals presenting rotational behavior were used to proceed with experiments. This behavior test was also performed to verify injury progression and treatment efficacy. For rotational tests, animals were intraperitoneally injected with apomorphine hydrochloride (Sigma-Aldrich) at a dose of 0.5 mg/kg (10 mg/ml, in 0.9% saline with 0.02% ascorbic acid). The number of contralateral rotations to 6-OHDA injection was recorded for 1 min, as described by Ferrazoli et al. (2017).

#### 6-OHDA Injury Progression

**Figure 1:**
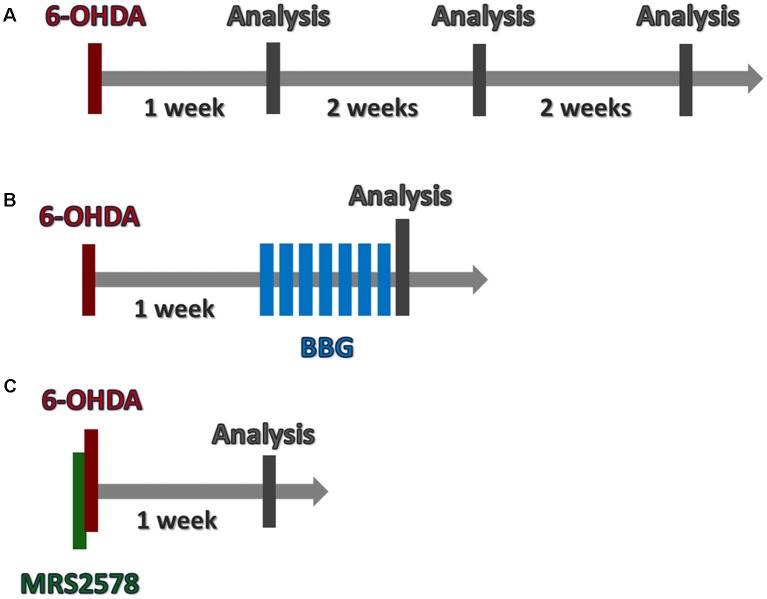
Timeline of *in vivo* procedures. **(A)** To analyze 6-hydroxydopamine (6-OHDA) lesion progression, animals were submitted to 6-OHDA injection. Brains were collected following 1, 3 or 5 weeks. **(B)** One week after 6-OHDA injection, animals were treated daily with Brilliant Blue G (BBG), during 7 days. Twenty-four hours later, brains were collected. **(C)** MRS2578 was injected 10 min prior 6-OHDA injection. After 1 week, brains were collected. **(A–C)** Rotational test was performed before brain collection.

In order to investigate the progression of 6-OHDA-induced injury, animals were submitted to rotational behavior test 1, 3 and 5 weeks after 6-OHDA injection. Animals were decapitated or perfused with phosphate-buffered saline (PBS) and 4% paraformaldehyde after 1, 3 or 5 weeks for sample extraction (Figure 1A).

#### Brilliant Blue G (BBG) Administration

In another set of experiments, 1 week after 6-OHDA injection, animals were submitted to BBG treatment. Daily, BBG (Sigma-Aldrich, 5–75 mg/kg in 0.9% saline with 0.02% ascorbic acid) or saline (control group) were intraperitoneally administered daily for 7 days. Animals were submitted to the rotational test and subsequently perfused for sample extraction (Figure 1B).

#### MRS2578 Administration

Ten-minutes prior to 6-OHDA injection, animals were injected with MRS2578 (Sigma-Aldrich, 2 μg/kg in 0.09% saline with 1% DMSO) or saline into the *striatum* with the following stereotactic coordinates from the Bregma: AP: −0, 4; ML: −3, 3; DV: −5, 2 (Paxinos and Watson, 2013). After 1 week, animals were perfused for sample extraction (Figure 1C).

### SH-SY5Y Cell Culture and Differentiation Into Dopaminergic Neurons

Human SH-SY5Y neuroblastoma cells were expanded in DMEM/F-12 medium (Gibco) supplemented with 10% fetal bovine serum (FBS), 100 U/ml penicillin (Sigma-Aldrich), 0.1 mg/ml streptomycin (Sigma-Aldrich) and 0.01 μM non-essential amino acids (Gibco). The cells were incubated at 37°C in 5% CO_2_. Medium was changed every 3 days. For dopaminergic differentiation, the cells were plated in 24-well plates at a density of 6 × 10^4^ cells per well. After 24 h, differentiation was induced by reducing the FBS concentration to 1% and supplementing the medium with 10 μM all trans-retinoic acid (Sigma-Aldrich). Cells were maintained in culture for 7 days at 37°C in 5% CO_2_, and the medium was changed every 2 days (Lopes et al., 2010).

#### Cell Viability Assay by Tetrazolium Reduction

In order to verify neuroprotective effects of MRS2578, SH-SY5Y cells differentiated into dopaminergic neurons were submitted to a cell viability assay based on measuring reduction of 3-(4,5-dimethylthiazol-2yl)-2,5-diphenyl tetrazolium (MTT) bromide to E, Z-1-(4,5-dimethylthiazol-2-yl)-1,3-diphenylformazan. Neurons were treated for 30 min with MRS2578 at concentrations of 1, 10 and 100 μM. After treatment, cell death was induced by 100 μM 6-OHDA. After 22 h, 1 mg/mL MTT was added for 2 h. The formazan crystals were dissolved in DMSO and the absorbance at 600 nm was analyzed on the FlexStation 3 Multi-Mode Reader Microplate ReaderFast instrument (Molecular Devices, San Jose, CA).

### Immunohistochemistry

For immunohistochemical analysis, each animal was deeply anesthetized with a ketamine/xylazine solution (100 mg/kg and 10 mg/kg, respectively) and perfused with approximately 400 ml phosphate-buffered saline using a peristaltic pump (World Precision Instruments, Sarasota, FL, USA). Subsequently, 200 ml of 4% paraformaldehyde in PBS (Sigma-Aldrich) solution at 4°C were used for tissue fixation. Brains were removed and incubated for 72 h in a 30% sucrose solution in PBS at 4°C for dehydration. Brains were placed in Tissue Tek^®^ medium for coronal sectioning in a cryostat (HM 500 OM, MICROM International GmbH, Walldorf, Germany) to obtain sequential sections of 30 μm. Slices were treated with 0.3% H_2_O_2_ (Labsynth, Diadema, Brazil) to eliminate endogenous peroxidase activity and incubated overnight with anti-tyrosine hydroxylase (dopaminergic neuron marker; Sigma-Aldrich, 1:500) or Iba-1 (microglial marker, Wako, Richmond, VA, USA, 1:500) primary antibodies for 24 h. Slices were washed with PBS and incubated with peroxidase-conjugated secondary antibodies (Jackson Laboratories, Bar Harbor, ME, USA) at a 1:200 dilution. The reaction was developed using 3,3′-Diaminobenzidine and VECTASTAIN^®^ Elite ABC HRP Kit (VectorLabs, Burlingame, CA, USA).

Images were obtained using the TissueFAXS tissue cytometer (TissueGnostics, Vienna, Austria). Optical density measurements of photomicrographs of tyrosine hydroxylase immunostained slices were performed to determine the density of TH^+^ fibers in the *striatum*. Measurements were made using grayscale and the mean values of three consecutive slices were used in each animal analysis. The values were normalized using the unlesioned control hemisphere as 100% to eliminate any difference in the background on each slice. In the *substantia nigra*, the number of TH-positive cells was counted using ImageJ software. Three consecutive slices obtained for each animal were counted. Mean values ± standard errors of the mean of injured and their respective control hemispheres are reported.

### Quantitative Real Time Polymerase Chain Reaction

Total RNA was extracted from *striatum* using the TRIzol Reagent (Invitrogen, Carlsbad, CA, USA) following manufacturer’s instruction. All samples were further treated with amplification grade DNase I (Sigma-Aldrich, St. Louis, MO, USA). Reverse transcription for cDNA synthesis was carried out on a thermal cycler using the RevertAid Reverse Transcriptase (Thermo Fisher Scientific, São Paulo, Brazil). First strand synthesis was done according to the manufacturer’s protocol (Invitrogen, Carlsbad, CA). RT-PCR was performed in 15 μl of reaction buffer containing of 1 μg cDNA, SYBR Green Master Mix (Life Technologies), and 5 pmol of each coding sequence-specific primers: for the P2X7 receptor—Fw, GGGAGGTGGTTCAGTGGGTAA, Rev, GGATGCTGTGATCCCAACAAA; for the P2Y6 receptor—Fw, CAGGATGTCTGCTGGAACCT; Rev, CCCTCTCAGCCTCAAGCTAC. Thermal cycling conditions consisted of a denaturation step for 10 min at 95°C followed by 40 cycles for denaturation for 15 s at 95°C, and annealing/extension for 1 min at 60°C, followed by melting curve analysis. The comparative 2^−ΔΔCT^ method was employed for relative quantification of gene expression as previously described (Pal et al., 2013) using *RPL0*—Fw, CTCGCTTCCTAGAGGGTGTCCGC; Rev, CTCCACAGACAAAGCCAGGAC—as internal standard for normalization, for each animal.

### Statistical Analysis

Statistical analyses were performed using Statistica software (version 7.0, StatSoft, Tulsa, OK). Rotational test, immunohistochemistry and qRT-PCR data were analyzed using two-way analysis of variance (ANOVA) followed by *post hoc* test. A (*p*)-value of 0.05 was assumed as significant. Shown results are mean values of at least three independent experiments. Error bars represent the mean ± standard error of the mean (SEM). All *F*- and *P*-values from *in vivo* experiments are presented in Supplementary Table S1.

## Results

### Time Kinetics of 6-OHDA Injury

In our rat model, no rotational behavior was observed before 6-OHDA injury of the nigrostriatal pathway. After 1, 3 and 5 weeks of 6-OHDA lesion, the number of rotations increased over time, as expected (Supplementary Figure S1). Striatal immunohistochemical staining of the injured brain hemispheres for tyrosine hydroxylase diminished after 1 and 5 weeks from 6-OHDA lesion compared to the respective unaffected control hemisphere (Supplementary Figures S1B,C). Moreover, in the *substantia nigra* the number of tyrosine hydroxylase^+^ neurons decreased in both analyzed time points when compared to non-injured control hemispheres (Supplementary Figures S1D,E).

### Purinergic *P2X7* and *P2Y6* Receptor Expression in 6-OHDA Lesion Progression

**Figure 2:**
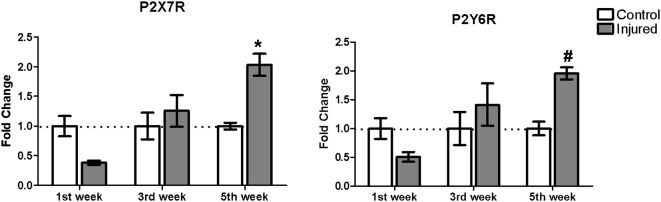
Gene expression of P2X7 and P2Y6 receptor subtypes in the *striatum* of rats following 1, 3 and 5 weeks after injury with 6-OHDA. Gene expression levels of the purinergic receptors curve in control and 6-OHDA-injured (7 μg/kg) hemispheres. *p < 0.05, in relation to respective control hemisphere. *F*_(2,12)_ = 10.6406, *p* = 0.002. ^#^*p* < 0.05, in relation to the injured hemisphere from the first week post injury, *F*_(2,12)_ = 5.6969, *p* = 0.018. *n* = 3 for each group. Two-way analysis of variance (ANOVA) followed by Bonferroni *post hoc* tests was used for statistical analysis.

We analyzed P2X7 receptor gene expression in animals submitted to 6-OHDA lesion, at three different time points: 1, 3 and 5 weeks after 6-OHDA injection. Besides normalizing receptor gene expression data with those of RPL0, that did not differ between experimental groups (Supplementary Figure S2), the fold change of purinergic receptors gene expression is shown in Figure 2 in relation to the respective control hemisphere. Five weeks after 6-OHDA injury, P2X7 receptor gene expression augmented by around two-fold in relation to the respective control hemisphere. Although P2Y6 receptor gene expression in the samples of 5 weeks post 6-OHDA injury did not present statistical difference in relation to the respective control hemisphere, increased fold change was present as compared to the injured hemisphere after 1 week post-lesion (Figure 2).

### Effects of P2X7 Receptor Inhibition by Chronic Brilliant Blue G Administration

**Figure 3:**
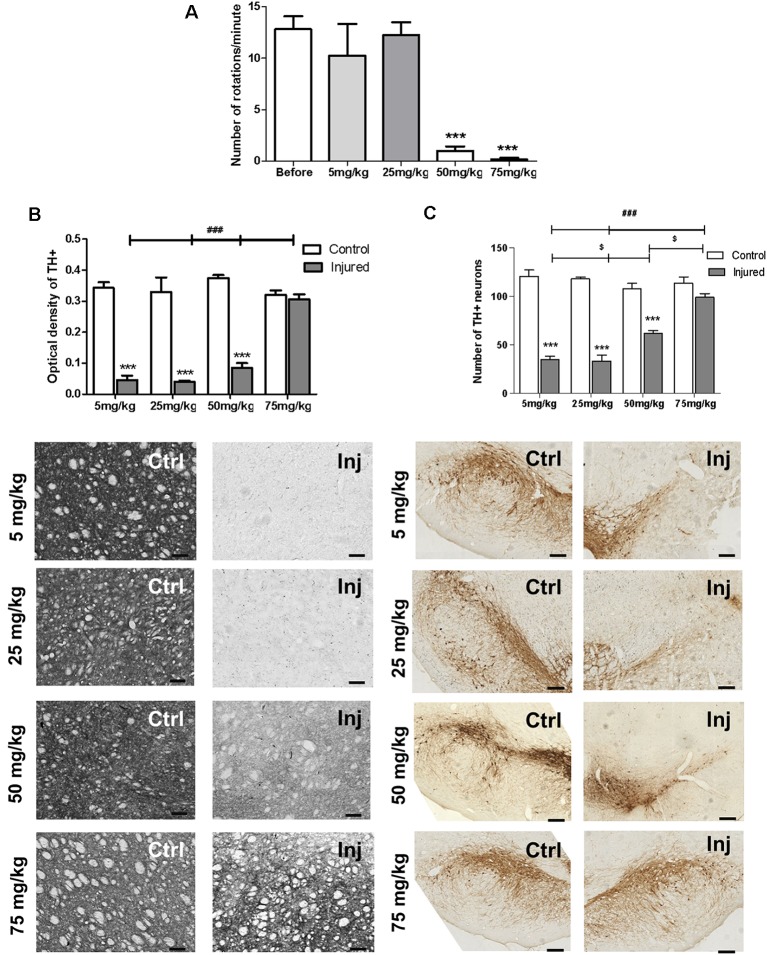
Neuroregenerative effects of BBG treatment in 6-OHDA-injured rats. BBG at 5, 25, 50 and 75 mg/kg doses was administered during 7 days starting 1 week after injury induction by 6-OHDA (7 μg/kg). **(A)** Animals were submitted to the apomorphine-induced rotational test before and after the treatment. ***p < 0.001 in relation to the number of pre-treatment rotations, *n* = 3–4; *F*_(4,21)_ = 23.8528, *p* < 0.001. One-way ANOVA followed by the Tukey *post hoc* test. Immunohistochemical analysis of tyrosine hydroxylase labeling of the *striatum* **(B)**, measured as optical density of staining; scale bar: 100 μm; *n* = 4 for BBG 5 and 25 mg/kg; *n* = 6 for BBG 50 and 75 mg/kg; *F*_(3,32)_ = 30.262, *p* < 0.001. Tyrosine hydroxylase labeling of the *substantia nigra* **(C)**, expressed by number of neurons, scale bar: 200 μm; *n* = 4 for BBG 5 and 25 mg/kg; *n* = 6 for BBG 50 and 75 mg/kg; *F*_(3,32)_ = 23.266, *p* < 0.001. Control (Ctrl) and injured (Inj) hemispheres. Data are expressed as mean ± standard errors of the mean. ***p < 0.001 in relation to the respective control hemisphere; ^$^*p* < 0.05 injured hemisphere from BBG 50 mg/kg in relation to other injured hemispheres; ^###^*p* < 0.001 injured hemisphere from BBG 75 mg/kg in relation to other injured hemispheres. Two-way ANOVA followed by the Bonferroni *post hoc* test.

Literature data show that preventive treatment of rats with BBG before lesion protected dopaminergic neurons from 6-OHDA-induced death (Carmo et al., 2014). Here, we tested the neuroregenerative effect of daily intraperitoneal injections of BBG treatment for 7 days. 6-OHDA injured animals treated with BBG at the dose of 50 and 75 mg/kg, but not at 5 and 25 mg/kg, decreased rotation behavior in the apomorphine-induced rotational test (Figure 3A). At the higher dose, 75 mg/kg, BBG treatment significantly reestablished striatal dopaminergic ramifications. The optical density of tyrosine hydroxylase staining of the injured hemisphere showed no difference compared to the control hemisphere (Figure 3B). In the *substantia nigra*, BBG at doses of 50 and 75 mg/kg restored tyrosine hydroxylase^+^-dopaminergic neurons, suggesting that neuroregeneration has occurred (Figure 3C). BBG at 75 mg/kg was used for further experiments.

**Figure 4:**
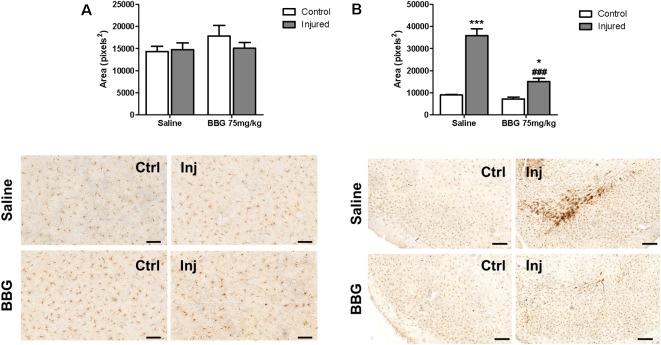
Microglial activation determined as Iba-1 labeling in hemispheres of BBG-treated and untreated animals. Animals were treated with 75 mg/kg daily for 7 days, 1 week after 6-OHDA- induced injury (7 μg/kg). **(A)** *Striatum*, scale bar: 100 μm; *F*_(1,20)_ = 0.900, *p* = 0.354; and **(B)** *substantia nigra*, scale: 200 μm; *F*_(1,20)_ = 28.274, *p* < 0.001. Areas labeled by Iba-1 immunostaining in control (Ctrl) and injured hemispheres (Inj) were subjected to densitometric analysis, and results are expressed as mean values ± standard errors of the mean. *p < 0.05, ***p < 0.001 in relation to respective control hemisphere; ^###^*p* < 0.001 in relation to injured hemisphere from saline group. *n* = 6 for each group. Two-way ANOVA followed by the Bonferroni *post hoc* test.

Microglial activation was studied as a possible target of BBG treatment at a dose of 75 mg/kg. 6-OHDA injection *per se* did not result in any alteration of striatal Iba-1 immunostaining (Figure 4A, injured/saline hemisphere), but increased Iba-1 immunostaining in the *substantia nigra* (Figure 4B, injured/saline group). This inflammatory effect was partially abolished by BBG treatment, as observed by decreased Iba-1 labeling compared to injured hemisphere of animals without treatment (Figure 4B). BBG treatment did not alter weight gain, pain behavior—as analyzed according to the Grimace pain scale—and survival rate (data not shown), as previously verified (Peng et al., 2009).

### P2Y6 Receptor Inhibition by MRS2578 Administration *in vivo*

**Figure 5:**
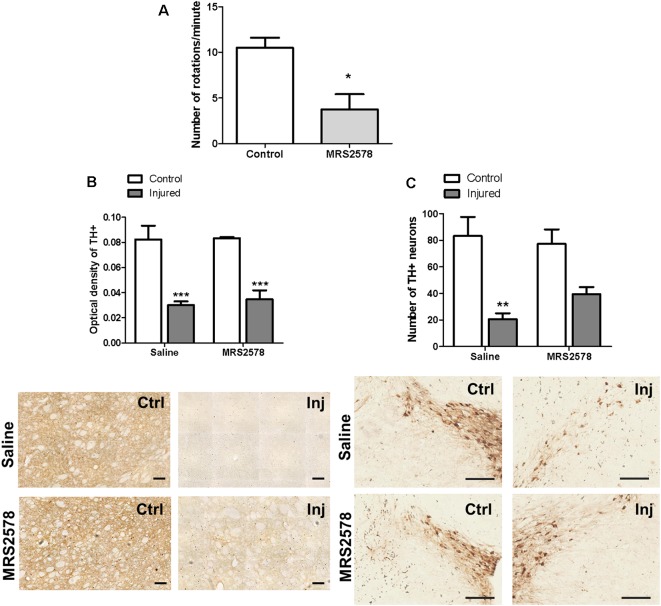
Effect of MRS2578 treatment on 6-OHDA-injured rats. **(A)** MRS2578 at a dose of 2 μg/kg was intrastriatally injected 10 min before (*n* = 8) or 5 weeks after (*n* = 5) the 6-OHDA at 7 μg/kg injection. Animals were submitted to the apomorphine-induced rotational test after 1 week. *p < 0.05 in relation to the number of pre-treatment rotations, *F*_(2,18)_ = 4.299, *p* = 0.029. One-way ANOVA followed by the Tukey *post hoc* test. Immunohistochemical analysis of tyrosine hydroxylase labeling in the *striatum* **(B)**, measured as optical density, scale: 100 μm, *F*_(1,20)_ = 4.109, *p* = 0.056; and **(C)** in the *substantia nigra*, expressed by the number of neurons, scale: 200 μm; *F*_(1,20)_ = 7.751, *p* = 0.011. Control (Ctrl) and injured (Inj) hemispheres. Data are expressed as mean values ± standard errors of the mean. *** *p* < 0.001, **p < 0.01 in relation to the respective control hemisphere; *n* = 6 for each group. Two-way ANOVA followed by the Bonferroni *post hoc* test.

Rotational behavior induced by the administration of apomorphine observed in the control group was abolished in animals treated with a single dose of MRS2578 prior to injury with 6-OHDA, indicating a preventive antiparkinsonian effect. When administered 5 weeks after injury, MRS2578 treatment did not reduce rotational behavior (Figure 5A). Moreover, preventive administration of MRS2578 did not protect against the decrease of striatal dopaminergic ramifications (Figure 5B), since the optical density of injured hemispheres was less than those of respective control hemispheres. Differently, in the *substantia nigra*, this treatment with MRS2578 at least partially prevented the decrease in the number of dopaminergic neurons in the injured hemisphere, as compared to the injured hemisphere from animals treated with saline (Figure 5C).

**Figure 6:**
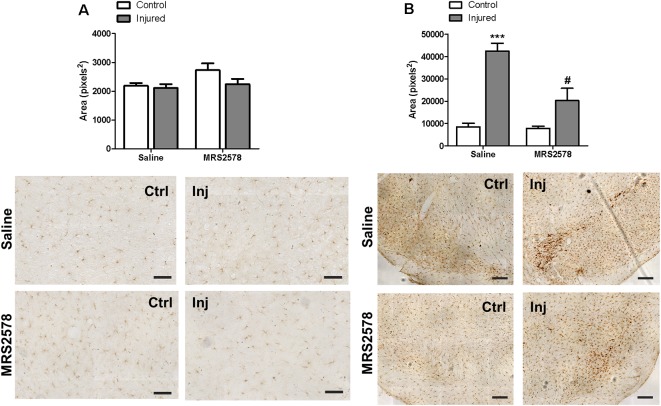
Immunohistochemical analysis of Iba-1 labeling in striatum and substantia nigra of animals treated by intrastriatal injection of 2 μg/kg MRS2578 10 min prior to 6-OHDA injection. **(A)** *Striatum*, scale: 100 μm, *F*_(1,20)_ = 1.276, *p* = 0.272; and **(B)** *substantia nigra*, scale: 200 μm, *F*_(1,20)_ = 26.7926, *p* < 0.001. The area marked with Iba-1 in the control (Ctrl) and injured hemispheres (Inj) are expressed as mean ± standard error of the mean (SEM). *n* = 6 for each group. ***p < 0.001, in relation to the respective control hemisphere; ^#^*p* < 0.05 in relation to injured hemisphere from saline group. Two-way ANOVA followed by the Bonferroni *post hoc* test.

No changes in Iba-1 microglial marker expression were detected in the striatal region of any group (Figure 6A). On the other hand, preventive treatment with MRS2578 attenuated microglial activation in the *substantia nigra* of injured hemispheres (Figure 6B).

### Effect of MRS2578 Treatment *in vitro*

**Figure 7:**
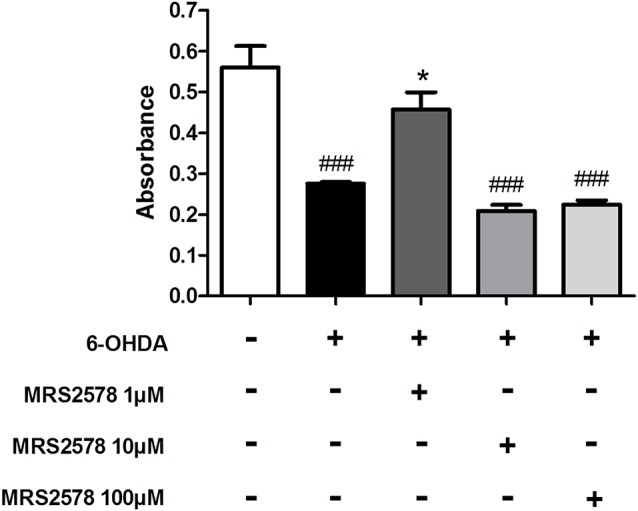
Cell viability after 6-OHDA insult. Cell viability of differentiated SH-SY5Y human cells in dopaminergic neurons, treated with MRS2578 (MRS) at doses of 1, 10 and 100 μM for 30 min and induced to death for 24 h by 6-OHDA (100 μM) was tested by the MTT reduction assay. Absorbance at 600 nm is expressed as mean ± standard errors of the mean. *p < 0.05 compared to group with 6-OHDA-induced death without treatment; ^###^*p* < 0.001 in relation to control group; *n* = 3. One-way ANOVA followed by the Bonferroni *post hoc* test.

To clarify whether the effects of MRS2578 treatment also involve inhibition of P2Y6 receptors expressed by dopaminergic neurons and subsequent neuroprotective action, we performed a cell viability assay. MRS2578 is known to be cytoprotective for SH-SY5Y neuroblastoma cells, a model of *in vitro* dopaminergic differentiation (Qian et al., 2018); however there are no reports of action against cell death induced by 6-OHDA. We showed here that treatment of SH-SY5Y cells differentiated into dopaminergic neurons with MRS2578 at 1 μM concentration, but not at 10 and 100 μM concentrations, prevented cell death induced by 6-OHDA (Figure 7).

## Discussion

Previous studies have shown implications of purinergic receptors in neurodegenerative diseases, including PD (reviewed by Oliveira-Giacomelli et al., 2018). We report here that antagonism of purinergic P2X7 or P2Y6 receptors present neuroregenerative or neuroprotective effects, respectively, in the 6-OHDA animal model of PD. Moreover, antagonism of these receptors reduced the microglial activation state in the *substantia nigra*.

Several 6-OHDA injury protocols have been reported using different doses, sites of injection, and extension of dopaminergic neuron death (Deumens et al., 2002). Using a previously established protocol by our group (Ferrazoli et al., 2017), we characterized the 6-OHDA-induced PD animal model, which is representative of dopaminergic neuron degradation occurring during the course of this disease (Deumens et al., 2002). Animals presented rotational behavior following 1 week of surgery, which persisted for 5 weeks of investigation. Immunohistochemical analysis of dopaminergic neurons positive for tyrosine hydroxylase from the *substantia nigra* and their ramifications in the *striatum* showed an extensive decrease in immunostaining in injured hemispheres compared to the respective control hemispheres, persisting for the investigated period of 5 weeks. These data indicate that, at least 5 weeks after lesion, animals did not present any spontaneous recovery, as observed in a range of 6-OHDA lesion variants, confirming the adequacy of injection coordinates (Schwarting and Huston, 1996). Dopamine depletion was previously reported to be accompanied by increased number of striatal TH-positive interneurons (Betarbet et al., 1997), but we did not detect any cell bodies stained for TH in the injured *striatum* (Supplementary Figure S3).

In order to verify possible changes in gene expression patterns of purinergic receptors and to identify possible targets for PD treatment, we analyzed striatal P2 purinergic receptor gene expression during 5 weeks after 6-OHDA-induced injury. The injured *striatum* of the 5th week after lesion presented augmented gene expression of P2X7 receptors in comparison to the respective control hemisphere. It is known that the sustained activation of the P2X7 receptor under pathological conditions may lead to the formation of membrane macropore with increased permeability that induces pro-apoptotic signaling cascades (Di Virgilio et al., 2018). In fact, the expression of the P2X7 receptor has been shown to be directly related to the capability of ATP to trigger apoptotic processes (North, 2002). These data corroborate the observed increase in *P2X7* receptor gene expression in 6-OHDA injured hemispheres. The intense release of ATP from damaged cells could result in P2X7 receptor-induced membrane macropore formation and activation of microglial cells with increased release of proinflammatory cytokines (Monif et al., 2010). Controversially, Amadio et al. (2007) showed that P2X7 receptor expression is not altered in *substantia nigra* and *striatum* after 1.5 months of 6-OHDA injury. We speculate that *P2X7* receptor gene expression could be peaking between 5 weeks and 1.5 months after 6-OHDA injection, but further experiments need to be performed to confirm this hypothesis.

Treatment with the P2X7 receptor antagonist BBG at a dose of 75 mg/kg, administered after dopaminergic neuron depletion, reestablished the nigrostriatal pathway, as observed by tyrosine hydroxylase staining. Previous studies showed therapeutic effects of P2X7 receptor blockade in animal models of PD. The selective antagonism of P2X7 receptor by intrastriatal injections of A-438059 (1 h before and 1 h after 6-OHDA injection) prevented striatal dopaminergic deficit induced by 6-OHDA (Marcellino et al., 2010). A study from Carmo et al. (2014) showed that BBG treatment in a preventive protocol, i.e., before dopaminergic depletion, protected dopaminergic neurons in the *substantia nigra* against 6-OHDA–induced neuronal death. BBG at a dose of 45 mg/kg was administered daily for 14 days, the first dose 2 h before injury, and prevented rotational behavior, short-term memory impairment and dopaminergic deficit in the *striatum* and *substantia nigra* (Carmo et al., 2014). In the LPS-induced PD rat model, BBG treatment at a dose of 50 mg/kg, injected during 15 days at the same time as LPS injections, prevented death of nigral dopaminergic neurons death as well as microglial activation (Wang et al., 2017). Our group previously demonstrated that daily administration of BBG (50 mg/kg, intraperitoneal) for 7 days, starting 1 week after the induction of cell death, also partially restored the number of cell bodies of the *substantia nigra*, inhibiting the rotational behavior induced by apomorphine (Ferrazoli et al., 2017). However, striatal ramifications were not reestablished at this BBG concentration. Here, we show that treatment with a higher dose of 75 mg/kg BBG both restored dopaminergic neuron number in the *substantia nigra* and dopaminergic ramifications in the *striatum*, thus providing more prominent therapeutic effects.

Immunohistochemical staining for Iba-1 microglial marker showed that BBG treatment at a dose of 75 mg/kg restored the inactive state of microglia cells in the *substantia nigra*, such as observed in the absence of the 6-OHDA lesion. In animal models, the release of ATP due to 6-OHDA-induced cell death amplified neuroinflammation and injury and increased the release of proinflammatory cytokines, such as IL-1β, transforming growth factor (TNF)-α, and nitric oxide (Zhang et al., 2005). Part of this action occurs due to ATP binding and activation of P2X7 receptors. In agreement, Melani et al. (2006) showed that P2X7 receptor expression is increased in reactive microglia cells in an animal model of ischemia. Literature data show that P2X7 receptor antagonism induces neuroprotection associated with reduced microglial activity and inflammatory responses (Durrenberger et al., 2012). Recently, Grygorowicz and Strużyńska (2019) demonstrated that BBG reduces microglia activation in a rat model of autoimmune encephalomyelitis. Thus, our results indicate that the P2X7 receptor is involved in microglia activation and the neuroinflammatory state induced by 6-OHDA injury.

Overall, inhibition of the P2X7 receptor by BBG treatment could result in reduced immune responses in the lesion microenvironment, preventing lesion exacerbation and creating favorable conditions to neuronal repopulation by tissue repair mechanisms. As reviewed by Oliveira et al. (2016), neural progenitor cells in the subventricular and subgranular areas of the adult brain express P2X7 receptors (Genzen et al., 2009; Messemer et al., 2013), and the inhibition of this receptor is essential for the differentiation of neural precursor cells into neurons *in vitro* (Glaser et al., 2014). Although further experiments should be performed to confirm this hypothesis, P2X7 receptor antagonism by BBG could be inducing differentiation and migration of neural progenitor cells from neurogenic areas of the brain towards the injury site. This hypothesis is corroborated by a study showing that injection of TNF-α induces migration of SVZ precursors towards the *striatum* (de Chevigny et al., 2008). Further, neurogenesis has also been shown for adult mouse in the *substantia nigra* (Zhão et al., 2003).

In addition to antagonizing P2X7 receptors, the BBG compound also blocks, with less potency, neuronal voltage-dependent sodium channels (Jo and Bean, 2011). It is known that an increase in membrane permeability to sodium contributes to axonal neurodegeneration (Stys et al., 1992). Moreover, voltage-dependent sodium channel blockade has been postulated to contribute to neuroprotective effects in different neurodegenerative diseases (Kapoor, 2008; Li et al., 2014; Verleye et al., 2016). P2X4 receptors are also antagonized by BBG (Ase et al., 2015). However, P2X4 receptor gene expression did not present any alterations in the 6-OHDA model of PD (Supplementary Figure S4) and, contrary to BBG, did not protect SH-SY5Y cells from 6-OHDA-induced cell death (Supplementary Figure S5).

Hracskó et al. (2011) demonstrated that P2X7 receptor knockout mice did not present any protective effects after MPTP-induced injury. Moreover, *P2X7* receptor gene expression increased after 1 week of this injury. While this data does not corroborate with our results, this study analyzed a single timepoint after injury, i.e., 1 week. This could be intrinsic to the different used models, once this protocol of MPTP injection predominantly induces necrotic cell death. Moreover, this could be due to the different used species, since MPTP does not induce any significant dopaminergic degeneration in rats (Jackson-Lewis et al., 1995; Schmidt and Ferger, 2001; Schober, 2004).

In addition to the P2X7 receptor, *P2Y6* receptor gene expression gradually augmented in injured hemispheres 5 weeks after 6-OHDA injection, when compared to expression rates found in injured hemispheres following 1 week of injury. Implications of P2Y6 receptors have not yet been studied in animal models of PD. However, the P2Y6 receptor was shown to be involved in neuroinflammatory processes, and its inhibition protected dopaminergic neurons *in vitro* (Qian et al., 2018). A recent published analysis of peripheral blood mononuclear cells from patients with PD demonstrated an increase in P2Y6 receptor expression. The authors suggested that the P2Y6 receptor participates in the microglial activation and control of phagocytosis of viable neurons, and its inhibition prevents neuronal loss in inflammatory conditions (Neher et al., 2014; Yang et al., 2017). Taking into account that PD presents a neurodegenerative scenario accompanied by neuroinflammatory process, the increase in the P2Y6 receptor expression in the injured hemispheres could be due to the expansion of the inflammatory process.

We show here for the first time that injection with a single dose of the P2Y6 receptor antagonist MRS2578 before injury prevented the death of dopaminergic neurons in the 6-OHDA animal model. Our data corroborate *in vitro* studies carried out with SH-SY5Y cells, a model of dopaminergic differentiation, which upon treatment with MPP^+^ presented a peak in P2Y6 receptor expression, corresponding to the increase of proinflammatory cytokines (Qian et al., 2018). In addition, gene expression knockdown of this receptor resulted in decreased expression of proteins involved in oxidative stress (Qian et al., 2018), indicating a possible mechanism by which MRS2578 may be protecting 6-OHDA-induced death *in vitro*. However, the effect observed *in vivo* seems to be related to the modulation of microglial cell activation, as observed *in vitro* by Qian et al. (2018).

In the absence of BBG treatment, reactive microglial cells were detected in the *substantia nigra*, but not in the *striatum* of hemispheres injured with 6-OHDA. The observed results may reflect the location of affected neurons, since cell bodies of dopaminergic neurons of the nigrostriatal pathway are only present in the *substantia nigra*, and, therefore are highly susceptible to exacerbation of inflammatory processes and sensitive to induction of neurotoxicity (Kim et al., 2000). Moreover, microglial activation apparently depends on the injection site of 6-OHDA site. While here the 6-OHDA was injected in the medial forebrain bundle, studies based on *striatal* injection revealed induced reactive microglia also in the striatal region (Chadi and Gomide, 2004; Cheng et al., 2018).

Microglial is suggested to play a pivotal role in the 6-OHDA model. Recruitment and activation of resident microglia exacerbate secretion of proinflammatory cytokines and contribute to increased neuronal damage (Fuxe et al., 2008). Hereby observed neuroregenerative/neuroprotective effects of P2X7/P2Y6 receptors were accompanied by inhibition of microglial activation. Recently, the presence of microglial cells was observed exerting the process of phagoptosis, where these cells phagocyted viable neurons under neuroinflammatory conditions (Brown and Neher, 2014). In a murine model with 6-OHDA lesion, the presence of reactive microglial cells preceded neuronal loss and early phagocytosis of dopaminergic neurons (Marinova-Mutafchieva et al., 2009). UDP released by degenerating cells induced nucleotide-dependent microglial phagocytosis (Inoue et al., 2009). In culture of neurons and microglial cells, the use of the P2Y6 receptor antagonist, MRS2578, delayed LPS-induced neuronal death possibly by inhibiting microglial phagoptosis (Neher et al., 2014). Thus, this process could be a possible target for neuroprotective effects of P2Y6 receptor antagonism. P2Y6 receptor antagonism in SH-SY5Y cells prevented cell death induced by 6-OHDA, showing that, at least partially, neuroprotective effect of MRS2578 involves neuronal P2Y6 receptors. Accordingly, Qian et al. (2018) showed that P2Y6 receptor antagonism in SH-SY5Y cells prevented cell death induced by MPTP through antioxidant effect by inhibiting ERK and p38 phosphorylation. We observed a dose-dependent effect of P2Y6 receptor antagonism. Since MRS2578 irreversibly inhibits P2Y6 receptors, high doses of this antagonist could be blocking physiological activities of neuronal P2Y6 receptor. Corroborating this hypothesis, P2Y6 receptor activation exhibited antiapoptotic effect in astrocytes (Kim et al., 2003).

In conclusion, 6-OHDA-induced neurodegeneration, resulting in the death of dopaminergic neurons in *striatum* and *substantia nigra*, prompts the extensive release of ATP and UDP, which supposedly stimulate microglial purinergic receptors, resulting in microglial activation. Reduced Iba-1 staining let to suggest that P2X7 receptor antagonism reduces microglial activation. Exacerbated activation of the P2X7 receptor might promote microglial proliferation and release of cytokines, nitric oxide and reactive oxygen species that culminate in increased cell death induction. In addition, it induces the release of chemokines that recruit resident microglia. The neurodegeneration process results in the release of nitric oxide and cytokines, which stresses viable neurons. These cells increase UDP release, supposedly activating P2Y6 receptors expressed by reactive microglia and possibly induce the process of phagoptosis.

## Data Availability Statement

All datasets generated for this study are included in the article/Supplementary Material.

## Ethics Statement

This study was carried out in accordance with the principles of the Basel Declaration and recommendations of Guidelines of the Brazilian College of Animal Experimentation and NIH Guide for Care and Use of Laboratory Animals. The protocol was approved by the local ethics’ committee (certificates 15/2013, 04/2014, 57/2017).

## Author Contributions

ÁO-G: conception and design of the work, animal procedures, immunohistochemistry and RT-PCR, data analysis and interpretation, and manuscript elaboration. CA: animal procedures, immunohistochemistry, and manuscript elaboration. HS: animal procedures and manuscript revision. JC-V: RT-PCR, data analysis and interpretation, and manuscript revision. AJ: *in vitro* procedures, data analysis and interpretation, and manuscript revision. JB: *in vivo* procedures, data analysis and interpretation, and manuscript revision. HU: conception and design of the work, data interpretation, manuscript writing, and critical article review for important intellectual content.

## Conflict of Interest

The authors declare that the research was conducted in the absence of any commercial or financial relationships that could be construed as a potential conflict of interest.

## Abbreviations

6-OHDA: 6-hydroxydopamine
ATP: adenosine -5′-triphosphate
BBG: Brilliant Blue G
DAMP: Danger Associated Molecular Patterns
MPP^+^: 1-methyl-4-phenylpyridinium
UDP: uridine-5′-diphosphate.
