# Supplementary material for: P2Y6 and P2X7 Receptor Antagonism Exerts Neuroprotective/ Neuroregenerative Effects in an Animal Model of Parkinson’s Disease
Source: Front Cell Neurosci. 2019 Nov 8;13:476. doi: 10.3389/fncel.2019.00476 (PMC6856016; doi:10.3389/fncel.2019.00476)
Supplement: Supplementary file 1 [file Data_Sheet_1.PDF]

**Table 1.: F and p values from *in vivo* experiments.** F and P values from each variable analyzed (6-OHDA injury, BBG or MRS2578 treatment and time points) or from the interaction between two variables. RT-PCR and immunohistochemical results analyzed by two-way ANOVA followed by Bonferroni *posthoc* test. Rotational test results analyzed by one-way ANOVA followed by Tukey *posthoc* test.

| **Experiment** | | **6-OHDA injury** | **Treatment / Time** | **Interaction** |
|---|---|---|---|---|
| Gene Expression | P2X7 | F(1,12)=0.224, p=0.152 | F(2,12)=10.640, p=0.002 | F(2,12)=10.640, p=0.002 |
| | P2Y6 | F(1,12)=2.73, p=0.125 | F(2,12)=5.697, p=0.018 | F(2,12)=5.697, p=0.018 |
| BBG | Tyrosine Hydroxylase (*Striatum*) | F(1,32) =267.887, p=0.000 | F(3,32) = 19.738, p=0.000 | F(3,32) = 30.262, p=0.000 |
| | Tyrosine Hydroxylase (*Substantia nigra*) | F(1,32)=253.367, p=0.000 | F(3,32)=16.565, p=0.000 | F(3,32)=23.266, p=0.000 |
| | Iba-1 (*Striatum)* | F(1,20)=0.471, p=0.500 | F(1,20)=1.298, p=0.268 | F(1,20)=0.900, p=0.354 |
| | Iba-1 (*Substantia nigra*) | F (1,20) = 93.968, p=0.000 | F(1,20) = 39.844, p=0.000 | F(1,20) = 28.274, p=0.000 |
| | Rotational Test | | F(4,21)=23.853, p=0.000 | |
| MRS2578 | Tyrosine Hydroxylase (*Striatum*) | F(1,20)=0.013, p=0.000 | F(1,20)=0.1445, p=0.708 | F(1,20)=4.109, p=0.56 |
| | Tyrosine Hydroxylase (*Substantia nigra*) | F (1,20) = 77.422, p=0.000 | F (1,20) = 3.803, p=0.065 | F (1,20) = 7.751, p=0.011 |
| | Iba-1 (*Striatum*) | F(1,20)=2.997, p=0.099 | F(1,20)=4.404, p=0.048 | F(1,20)=1.276, p=0.272 |
| | Iba-1 (*Substantia nigra*) | F (1,20) = 90.578, p=0.000 | F(1,20) = 32.129, p=0.0015 | F(1,20) = 26.7926, p=0.000 |
| | MRS2578 rotational test | | F(2,18)=4.299, p=0.029 | |
