# Supplementary material for: P2Y6 and P2X7 Receptor Antagonism Exerts Neuroprotective/ Neuroregenerative Effects in an Animal Model of Parkinson’s Disease
Source: Front Cell Neurosci. 2019 Nov 8;13:476. doi: 10.3389/fncel.2019.00476 (PMC6856016; doi:10.3389/fncel.2019.00476)
Supplement: Supplementary file 2 [file Data_Sheet_2.pdf]

Universidade de São Paulo
Instituto de Química

Departamento de Bioquímica

Dr. Henning Ulrich
Full Professor

The Guest Editors of the Special Issue " Purinergic Signaling in Health and Disease" of Frontiers in Cellular Neuroscience
Profs. Eric Boué-Grabot, David Blum and Stefania Ceruti

São Paulo, September 15, 2019

Dear Drs. Eric Boué-Grabot, David Blum and Stefania Ceruti:

Please, find enclosed the resubmission of manuscript "P2Y6 and P2X7 receptor antagonism exerts neuroprotective/ neuroregenerative effects in an animal model of Parkinson's disease" by Ágatha Oliveira-Giacomelli et al. for consideration of publication in the Special Issue "Purinergic Signaling in Health and Disease" of Frontiers in Cellular Neuroscience. This has been an invited submission and reports so far undiscovered neuroprotective properties of P2Y6 receptor antagonism in the 6OH-dopamine-induced animal of Parkinson's disease as well as further studying therapeutic effects of P2X7 receptor inhibition in this model.
All queries of Reviewer 1 were responded, and new data were included in the manuscript. We thank for the endorsements of Reviewers 2 and 3, indicating that the paper is ready for publication.
The authors declare that there are no conflicts of interest regarding the publication of this paper.

I am looking forward to your response,

Sincerely

H Ulrich

Henning Ulrich, Ph.D.
Professor
Institute of Chemistry-University of São Paulo
