# Supplementary figures and images for: P2Y6 and P2X7 Receptor Antagonism Exerts Neuroprotective/ Neuroregenerative Effects in an Animal Model of Parkinson’s Disease
Source: Front Cell Neurosci. 2019 Nov 8;13:476. doi: 10.3389/fncel.2019.00476 (PMC6856016; doi:10.3389/fncel.2019.00476)

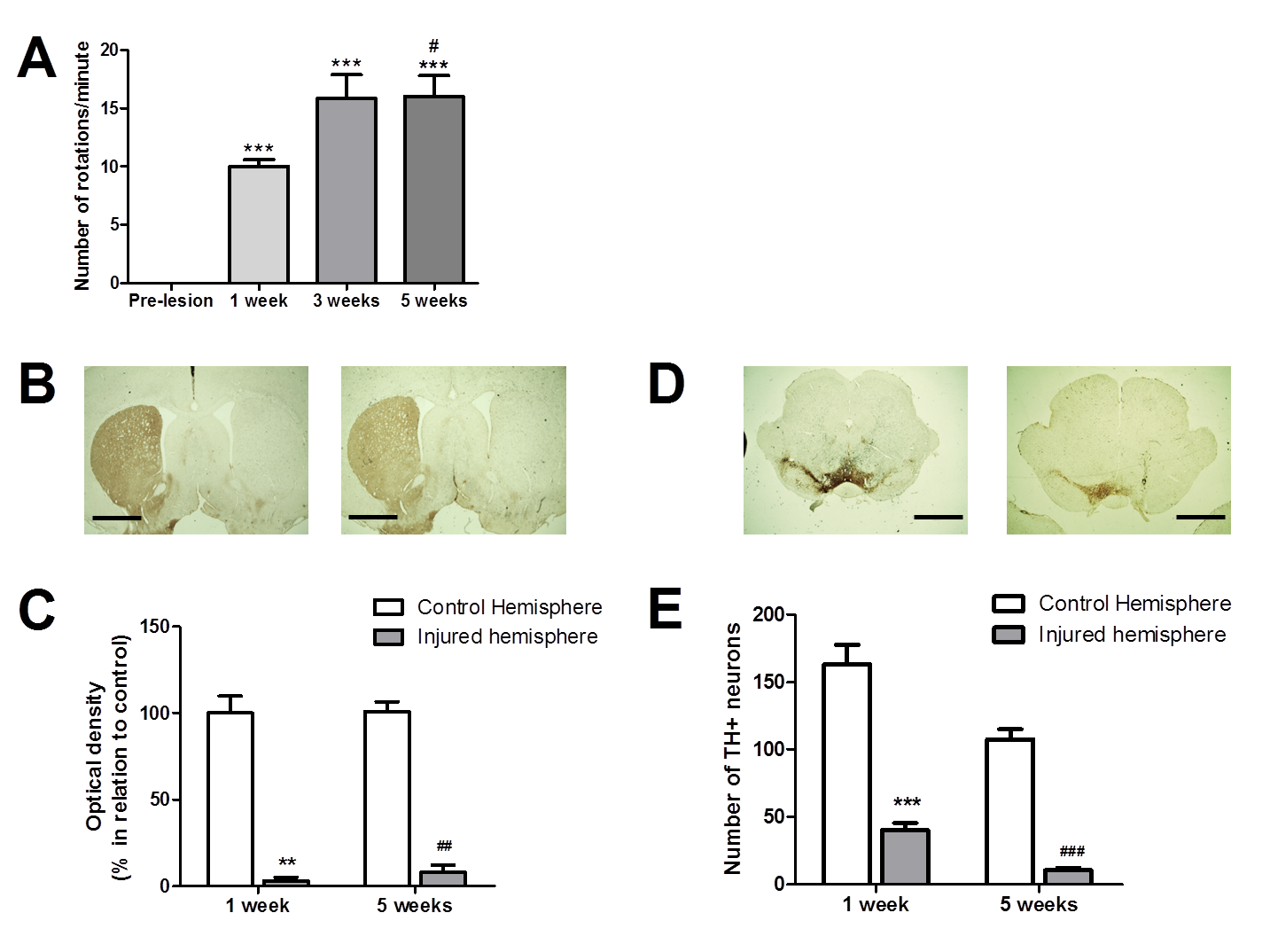

Supplement: Supplementary file 3 [file Image_1.TIF]

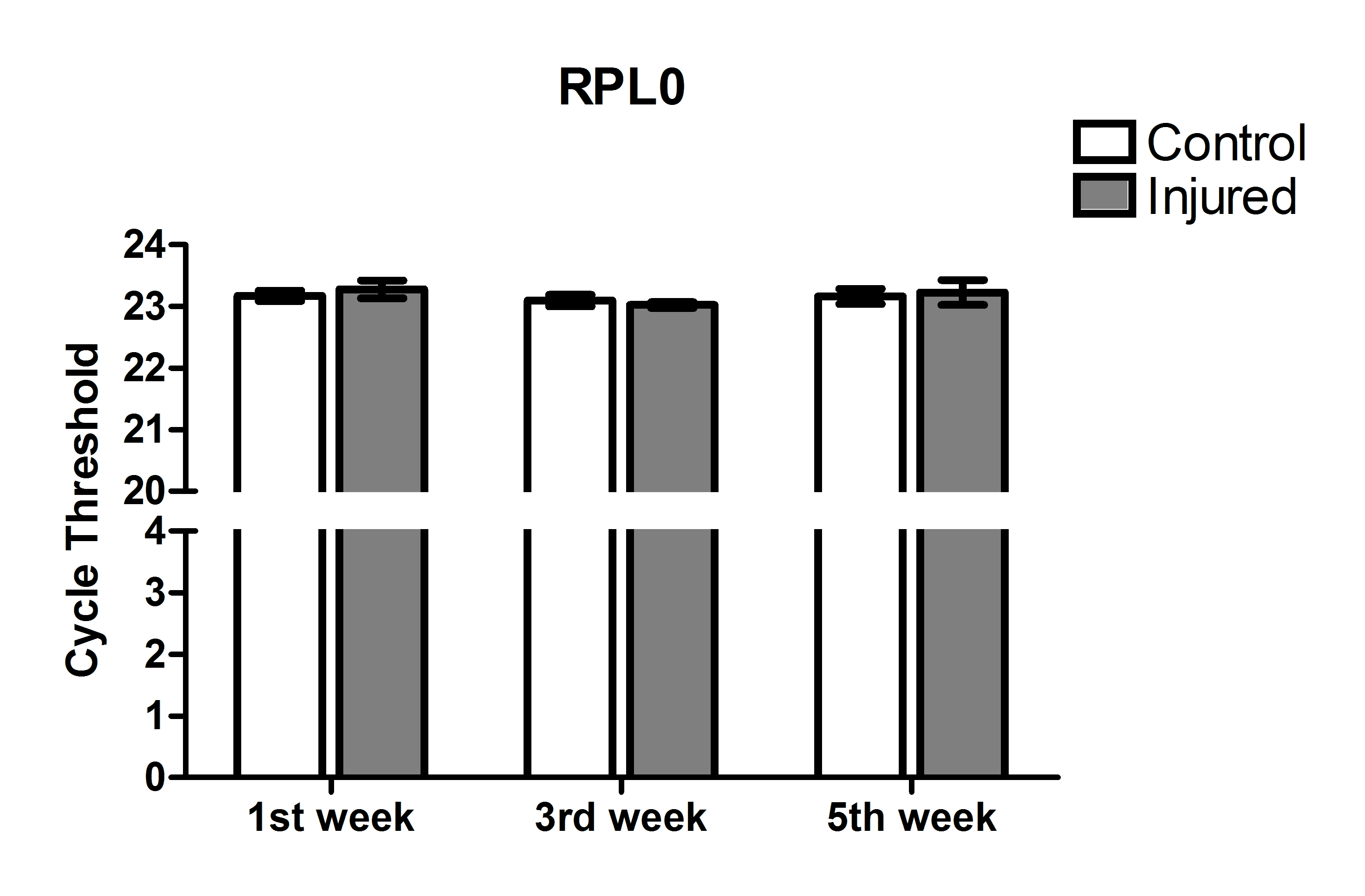

Supplement: Supplementary file 4 [file Image_2.TIF]

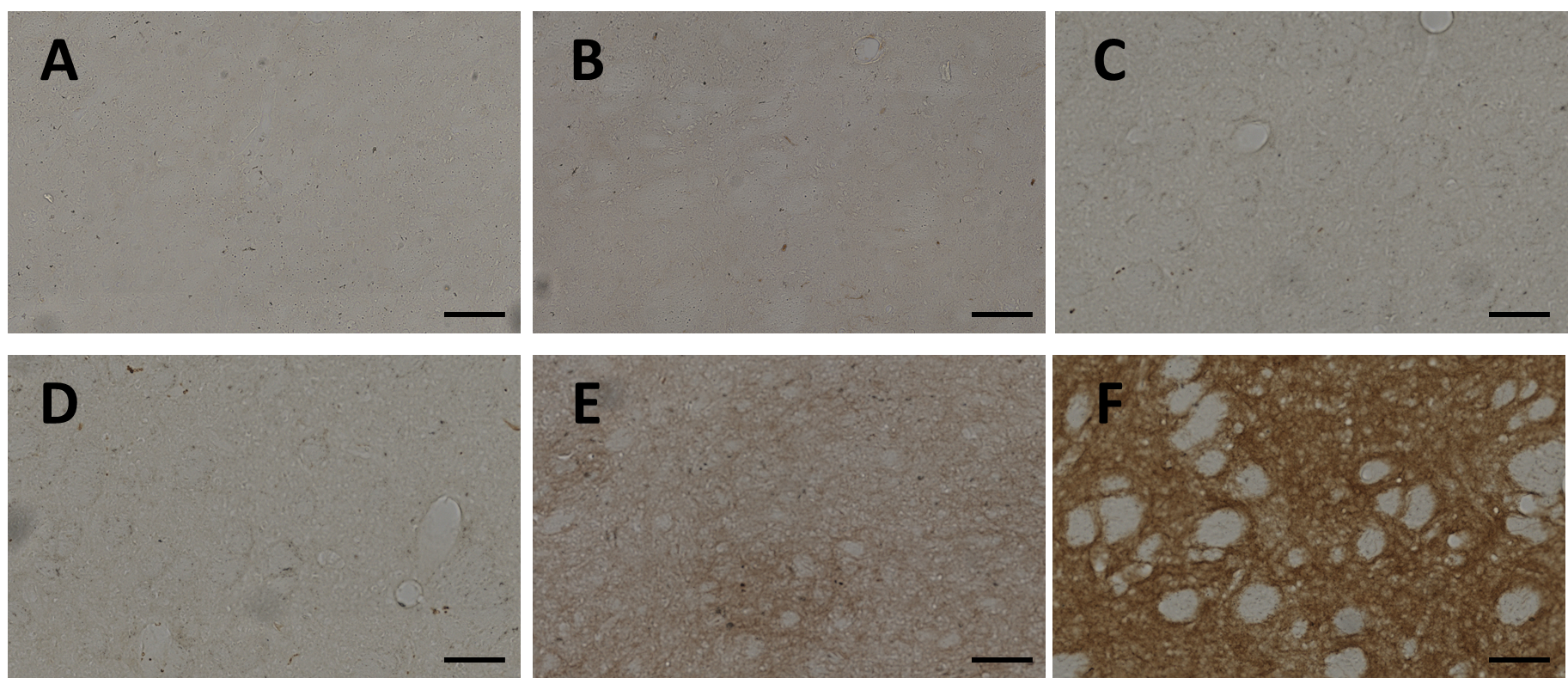

Supplement: Supplementary file 5 [file Image_3.TIF]

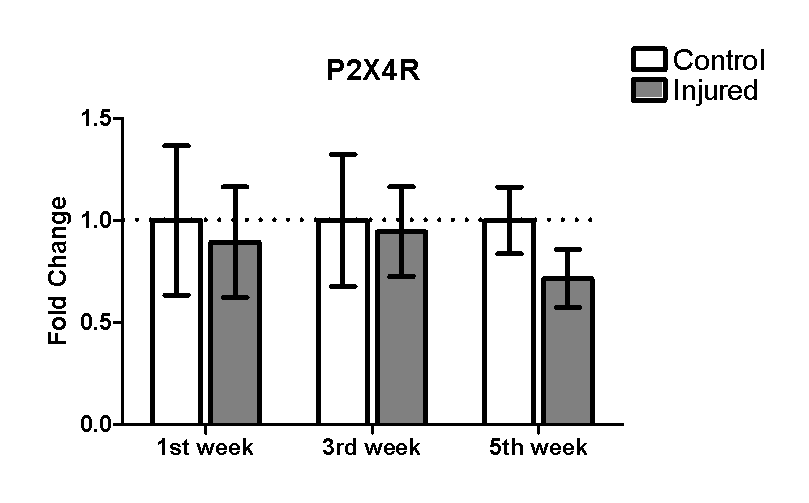

Supplement: Supplementary file 6 [file Image_4.TIF]

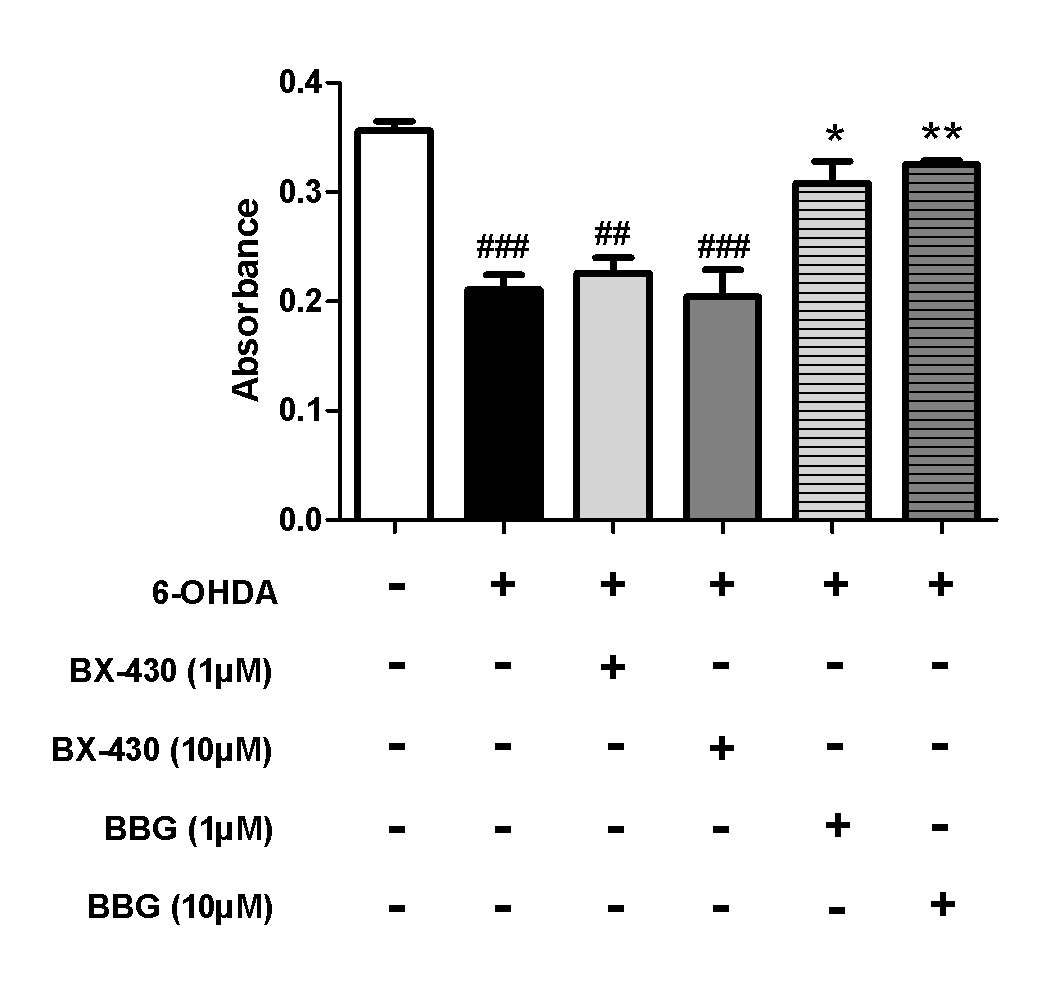

Supplement: Supplementary file 7 [file Image_5.TIF]
